# Supplementary material for: Thyroid Hormone Augmentation for Bipolar Disorder: A Systematic Review
Source: Brain Sci. 2022 Nov 14;12(11):1540. doi: 10.3390/brainsci12111540 (PMC9688441; doi:10.3390/brainsci12111540)
Supplement: Supplementary file 1 [file brainsci-12-01540-s001.zip › brainsci-2038879-supplementary.pdf]

Supplementary Materials

Table S1. Ovid. Database(s): APA PsycInfo 1806 to September Week 1 2022, EBM Reviews - Cochrane Central Register of Controlled Trials August 2022, EBM Reviews - Cochrane Database of Systematic Reviews 2005 to September 14, 2022, Embase 1974 to 2022 September 15, Ovid MEDLINE(R) and Epub Ahead of Print, In-Process, In-Data-Review & Other Non-Indexed Citations, Daily and Versions 1946 to September 15, 2022. Search Strategy:.

#	Searches	Results
1	exp Bipolar Disorder/	152734
2	exp bipolar depression/	54036
3	("Bipolar Affective Disorder*" or "bipolar affective psychos*" or "bipolar depression*" or "bipolar disorder*" or "bipolar illness*" or "Bipolar Mood Disorder*" or "bipolar psychos*" or Cyclothymia* or Cyclothymic or mania or manias or "Manic Depression*" or "manic depressive" or "manic disorder*" or "manic state*" or "manic-depressive psychos*" or "maniodepressive psychos*" or "mano depressive syndrome*").ti,ab,kf.	165741
4	1 or 2 or 3	206627
5	exp Thyroid Hormones/tu [Therapeutic Use]	11591
6	exp thyroid hormone/ and exp hormonal therapy/	2715
7	exp Thyroid Hormones/ and exp Hormone Therapy/	2745
8	(T3 or T4 or Cytomel or Euthyrox or levothyroxine or Levoxyl or liothyronine or Synthroid or Thyquidity or thyroxine or Tirosint or triiodothyronine or Triostat or Unithroid).ti,ab,kf.	269005
9	(5 or 6 or 7) and 8	5424
10	exp Triiodothyronine/	67096
11	exp Thyroxine/	107228
12	(((T3 or T4) and (hormone* or thyroid)) or Cytomel or Euthyrox or levothyroxine or Levoxyl or liothyronine or Synthroid or T4 or Thyquidity or thyroxine or Tirosint or triiodothyronine or Triostat or Unithroid).ti,ab,kf.	208977
13	9 or 10 or 11 or 12	255121
14	4 and 13	1325
15	(case* adj3 report*).mp,pt.	5584266
16	14 not 15	972
17	(exp animals/ or exp nonhuman/) not exp humans/	11937666
18	((alpaca or alpacas or amphibian or amphibians or animal or animals or antelope or armadillo or armadillos or avian or baboon or baboons or beagle or beagles or bee or bees or bird or birds or bison or bovine or buffalo or buffaloes or buffalos or "c elegans" or "Caenorhabditis elegans" or camel or camels or canine or canines or carp or cats or cattle or chick or chicken or chickens or chicks or chimp or chimpanze or chimpanzees or chimps or cow or cows or "D melanogaster" or "dairy calf" or "dairy calves" or deer or dog or dogs or donkey or donkeys or drosophila or "Drosophila	10581752

	melanogaster" or duck or duckling or ducklings or ducks or equid or equids or equine or equines or feline or felines or ferret or ferrets or finch or finches or fish or flatworm or flatworms or fox or foxes or frog or frogs or "fruit flies" or "fruit fly" or "G mellonella" or "Galleria mellonella" or geese or gerbil or gerbils or goat or goats or goose or gorilla or gorillas or hamster or hamsters or hare or hares or heifer or heifers or horse or horses or insect or insects or jellyfish or kangaroo or kangaroos or kitten or kittens or lagomorph or lagomorphs or lamb or lambs or lemur or lemurs or llama or llamas or macaque or macaques or macaw or macaws or marmoset or marmosets or mice or minipig or minipigs or mink or minks or monkey or monkeys or mouse or mule or mules or nematode or nematodes or octopus or octopuses or orangutan or "orang-utan" or orangutans or "orang-utans" or ostrich or ostriches or oxen or parrot or parrots or pig or pigeon or pigeons or piglet or piglets or pigs or porcine or primate or primates or quail or rabbit or rabbits or rat or rats or reptile or reptiles or rodent or rodents or ruminant or ruminants or salmon or sheep or shrimp or slug or slugs or swine or tamarin or tamarins or toad or toads or trout or urchin or urchins or vole or voles or waxworm or waxworms or wildlife or worm or worms or xenopus or "zebra fish" or zebrafish) not (human or humans or patient or patients)).ti,ab,hw,kf.	
19	16 not (17 or 18)	942
20	limit 19 to (editorial or erratum or note or addresses or autobiography or bibliography or biography or blogs or comment or dictionary or directory or interactive tutorial or interview or lectures or legal cases or legislation or news or newspaper article or overall or patient education handout or periodical index or portraits or published erratum or video-audio media or webcasts) [Limit not valid in APA PsycInfo,CCTR,CDSR,Embase,Ovid MEDLINE(R),Ovid MEDLINE(R) Daily Update,Ovid MEDLINE(R) PubMed not MEDLINE,Ovid MEDLINE(R) In-Process,Ovid MEDLINE(R) Publisher; records were retained]	20
21	19 not 20	922
22	remove duplicates from 21	626

Table S2. Scopus.

1	TITLE-ABS-KEY("Bipolar Affective Disorder*" or "bipolar affective psychos*" or "bipolar depression*" or "bipolar disorder*" or "bipolar illness*" or "Bipolar Mood Disorder*" or "bipolar psychos*" or Cyclothymia* or Cyclothymic or mania or manias or "Manic Depression*" or "manic depressive" or "manic disorder*" or "manic state*" or "manic-depressive psychos*" or "maniodepressive psychos*" or "mano depressive syndrome*")
2	TITLE-ABS-KEY(((T3 or T4) and (hormone* or thyroid)) OR Cytomel OR Euthyrox OR levothyroxine OR Levoxyl OR liothyronine OR Synthroid OR T4 OR Thyquidity OR thyroxine OR Tirosint OR triiodothyronine OR Triostat OR Unithroid)
3	1 and 2
4	TITLE-ABS-KEY(case* W/3 report*)
5	3 and not 4
6	TITLE-ABS-KEY((alpaca OR alpacas OR amphibian OR amphibians OR animal OR animals OR antelope OR armadillo OR armadillos OR avian OR baboon OR baboons OR beagle OR beagles OR bee OR bees OR bird OR birds OR bison OR bovine OR buffalo OR buffaloes OR buffalos OR "c elegans" OR "Caenorhabditis elegans" OR camel OR camels OR canine OR canines OR carp OR cats OR cattle OR chick OR chicken OR chickens OR chicks OR chimp OR chimpanze OR chimpanzees OR chimps OR cow OR cows OR "D melanogaster" OR "dairy calf" OR "dairy calves" OR deer OR dog OR dogs OR donkey OR donkeys OR drosophila OR "Drosophila melanogaster" OR duck OR duckling OR ducklings OR ducks OR equid OR equids OR equine OR equines OR feline OR felines OR ferret OR ferrets OR finch OR finches OR fish OR flatworm OR flatworms OR fox OR foxes OR frog OR frogs OR "fruit flies" OR "fruit fly" OR "G mellonella" OR "Galleria mellonella" OR geese OR gerbil OR gerbils OR goat OR goats OR goose OR gorilla OR gorillas OR hamster OR hamsters OR hare OR hares OR heifer OR heifers OR horse OR horses OR insect OR insects OR jellyfish OR kangaroo OR kangaroos OR kitten OR kittens OR lagomorph OR lagomorphs OR lamb OR lambs OR llama OR llamas OR macaque OR macaques OR macaw OR macaws OR marmoset OR marmosets OR mice OR minipig OR minipigs OR mink OR minks OR monkey OR monkeys OR mouse OR mule OR mules OR nematode OR nematodes OR octopus OR octopuses OR orangutan OR "orang-utan" OR orangutans OR "orang-utans" OR oxen OR parrot OR parrots OR pig OR pigeon OR pigeons OR piglet OR piglets OR pigs OR porcine OR primate OR primates OR quail OR rabbit OR rabbits OR rat OR rats OR reptile OR reptiles OR rodent OR rodents OR ruminant OR ruminants OR salmon OR sheep OR shrimp OR slug OR slugs OR swine OR tamarin OR tamarins OR toad OR toads OR trout OR urchin OR urchins OR vole OR voles OR waxworm OR waxworms OR worm OR worms OR xenopus OR "zebra fish" OR zebrafish) AND NOT (human OR humans or patient or patients))
7	5 and not 6
8	DOCTYPE(ed) OR DOCTYPE(bk) OR DOCTYPE(er) OR DOCTYPE(no) OR DOCTYPE(sh)
9	7 and not 8
10	INDEX(embase) OR INDEX(medline) OR PMID(0* OR 1* OR 2* OR 3* OR 4* OR 5* OR 6* OR 7* OR 8* OR 9*)
11	9 and not 10
